# Oxidative stress induces cortical stiffening and cytoskeletal remodelling in pre-apoptotic cancer cells

**DOI:** 10.15698/cst2025.08.310

**Published:** 2025-08-07

**Authors:** Aiman Jalmukhambetova, Aidana Baltabekova, Aizhan Tolebay, Nargiz Rakhimgerey, Ferdinand Molnár, Tri Thanh Pham, Agata N. Burska, Dos D. Sarbassov

**Affiliations:** 1 Department of Biology, School of Sciences and Humanities, Nazarbayev University, Astana, Kazakhstan.; 2 Department of Biomedical Sciences, School of Medicine, Nazarbayev University, Astana, Kazakhstan.; 3 National Laboratory Astana, Center for Life Sciences, Nazarbayev University, Astana, Kazakhstan.

**Keywords:** oxidative stress, cortical stiffness, atomic force microscopy, F-actin, arsenic trioxide and D form of vitamin C, cancer migration

## Abstract

An imbalanced production of reactive oxygen species (ROS) is linked to various aspects of cancer development, including cytoskeletal remodelling. However, the relationship between ROS, actin and cellular stiffness remains controversial. Here, we show that oxidative stress increases cortical stiffness in pre-apoptotic colon and pancreatic cancer cells via localized F-actin polymerization in the apical cortex — independent of changes in total F-actin levels. Using atomic force microscopy and flow cytometry, we demonstrate this effect across multiple ROS inducers: the combination of arsenic trioxide and *D*-enantiomer of vitamin C, hydrogen peroxide, and rotenone. These findings explain previously debated relationships on how ROS influence actin organization, which may affect cellular stiffness. By separating total from cortical actin effects, our study reveals a redox-sensitive mechanism that governs cytoskeletal remodelling and may impair cancer cell migration.

## Abbreviations

AFM - atomic force microscopy,

ATO - arsenic trioxide,

D-VC - D-enantiomere of vitamin C,

F-actin - filamentous actin,

LatB - Latrunculin B,

MFI - mean fluorescence intensity,

mtROS - mitochondrial ROS,

NAC - N-acetyl cysteine,

ROS - reactive oxygen species.

## INTRODUCTION

Metastasis and invasiveness are responsible for approximately 90% of cancer-related deaths 1. These traits depend on the ability of cancer cells to migrate through tissues, making cell migration a central feature of cancer aggressiveness and recurrence 2. One of the key biophysical properties influencing cell migration is cortical stiffness, which reflects the rigidity of actin cortex 3. Increased stiffness is generally associated with reduced deformability and slower migration in both cancer cells and cancer stem cells 4,5.

Cortical stiffness is commonly attributed to the organization and polymerization state of filamentous actin (F-actin). Actin polymerization at the leading edge and depolymerization at the rear facilitate directional movement 6. However, the relationship between actin dynamics and stiffness remains controversial. While some studies show that stress fiber formation and actin polymerization increase stiffness 7,8 others suggest that stiffness is modulated independently of actin organization— for example, through the assembly of cytoplasmic proteins such as metabolic enzymes, or via pH-induced cytosolic solidification 9,10.

F-actin dynamics and oxidative stress caused by an accumulation of reactive oxygen species (ROS) add another level of complexity. It is known that ROS can both promote and inhibit actin polymerization, depending on the context. Some studies suggest that ROS promote actin depolymerization through increased S-glutathionylation of the Cys374 residue, which is particularly susceptible to oxidation 11. In contrast other reports indicate that ROS can stimulate F-actin polymerization at the cell's leading edge through ERK-mediated recruitment of Arp2/3 complex, creating free-barbed ends of actin filaments 12,13. Yet, the net effect of ROS on cortical actin organization and stiffness, particularly under pre-apoptotic or therapeutic conditions, remains unclear.

In this study we used arsenic trioxide (ATO) and the *D*-enantiomer of vitamin C (D-VC), known as pro-oxidative agents that synergistically induce mitochondrial ROS and apoptosis in colorectal and pancreatic cancer cell lines and xenograft models 14,15. To assess the generalizability of oxidative stress effects on cellular mechanics, we employed two distinct cancer cell lines: AK 192 (mouse pancreatic) and HCT 116 (human colon). Differences in species and tissue origin allowed us to identify core cellular responses to oxidative stress that extend beyond a single cellular context. While their cytotoxic effects have been well studied, the impact of this oxidative stress on the cytoskeleton and biophysical properties of surviving or pre-apoptotic cells remains unknown.

Here we demonstrate that ROS increases cortical stiffness of pre-apoptotic cells, which is mediated by localized F-actin polymerization in the apical cortex and independent of changes in total F-actin levels. These findings clarify a previous controversy between ROS and actin mechanics as well as actin and cellular stiffness relationships, suggesting a redox-sensitive mechanism that regulates biophysical properties of cell cytoskeleton.

## RESULTS

### ATO/D-VC treatment synergistically induces pre-apoptotic phenotypes in cancer cells

**Figure 1:**
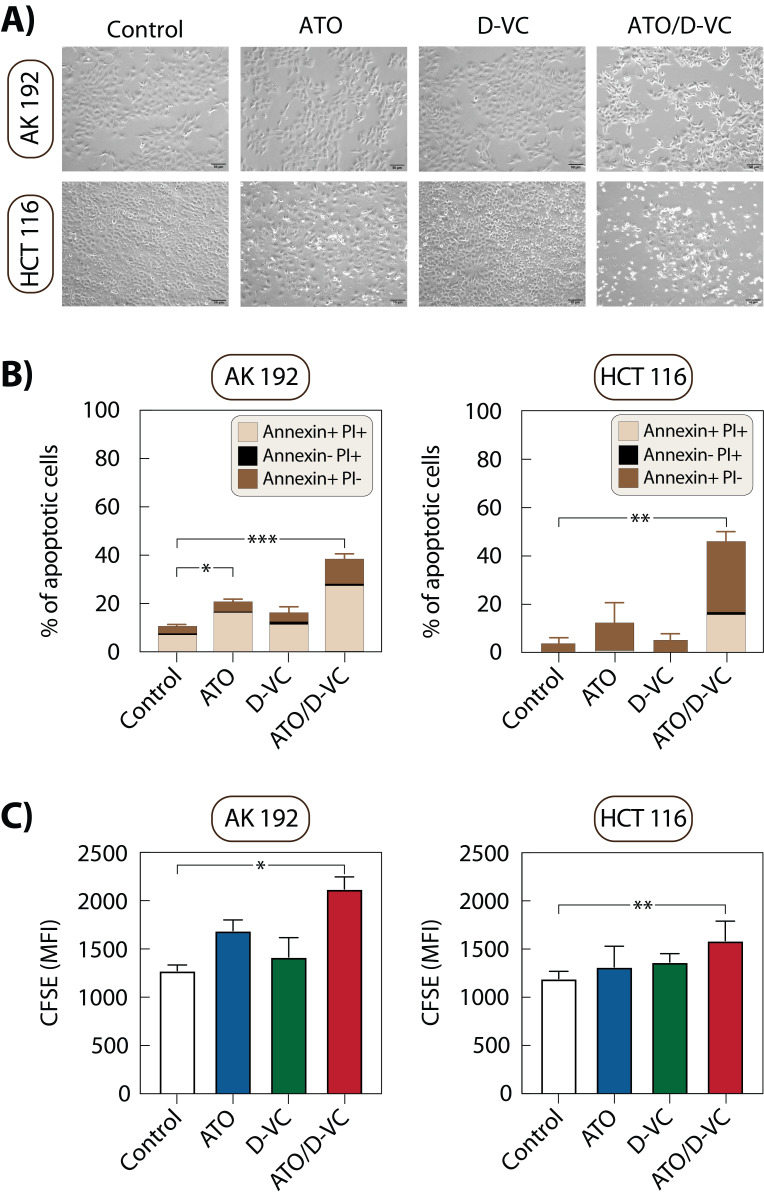
FIGURE 1: Effect of ATO and D-VC treatment on cell morphology, apoptosis and proliferation. **(A)** Bright field images of AK 192 and HCT 116 cells at 24 h and 72 h post-treatment; 10X magnification. **(B)** Quantitative analysis of flow cytometric apoptosis assay using staining with Annexin V and propidium iodide (PI) staining. Data represent the mean ± SD from three independent experiments. Cells positive for Annexin V and PI are classified as late apoptotic; Annexin V-positive and PI-negative as early apoptotic; and Annexin V-negative and PI-positive as necrotic. **(C)** Flow cytometric analysis of cell proliferation with carboxyfluorescein succinimidyl ester (CFSE) staining. Results are shown as mean fluorescence intensity (MFI); higher MFI indicates lower proliferation due to reduced dye dilution. Stars indicate statistical significance: p<0.05 (*), p<0.01 (**) and p<0.001 (***).

Microscopic examination of cell morphology showed shrinkage and detachment of AK 192 ells after 24 h and HCT 116 cells after 72 h of treatment with the cytotoxic oxidative ATO/D-VC drug combination, compared to control cells cultured in DMEM without treatment for the same length of time (**Fig. 1A**). A single drug treatment with either 5 µM ATO or 1 mM D-VC in AK 192 cells and 7.5 µM of ATO and 1.25 mM of D-VC in HCT 116 did not affect the shape and appearance of cancer cells.

Flow cytometry using Annexin V and propidium iodide (PI) staining demonstrated an increase in apoptotic populations in both cell lines following ATO/D-VC treatment: 37.9% in AK 192 cells after 24 h and 46.6% in HCT 116 cells after 72 h. In AK 192 cells, the majority were late apoptotic (27.4%), with a smaller proportion of early apoptotic cells (9.8%). In contrast, HCT 116 cells exhibited predominantly early apoptotic (29.8%) and fewer late apoptotic cells (15.1%). Single-drug treatments resulted in apoptosis levels similar to controls, with only a slight increase under ATO treatment (20.5% in AK 192 cells and 12.9% in HCT 116 cells) (**Fig. 1B**).

Further flow cytometry with CFSE staining was performed to assess cell proliferation. ATO/D-VC treatment decreased proliferation by 1.7-fold in AK 192 cells (mean fluorescence intensity (MFI): control 1276 vs. treated 2121.3) and by 1.3-fold in HCT 116 cells (MFI: control 1195.8 vs. treated 1588).

### The ATO/D-VC drug combination treatment impairs the migration rate of cancer cells

**Figure 2:**
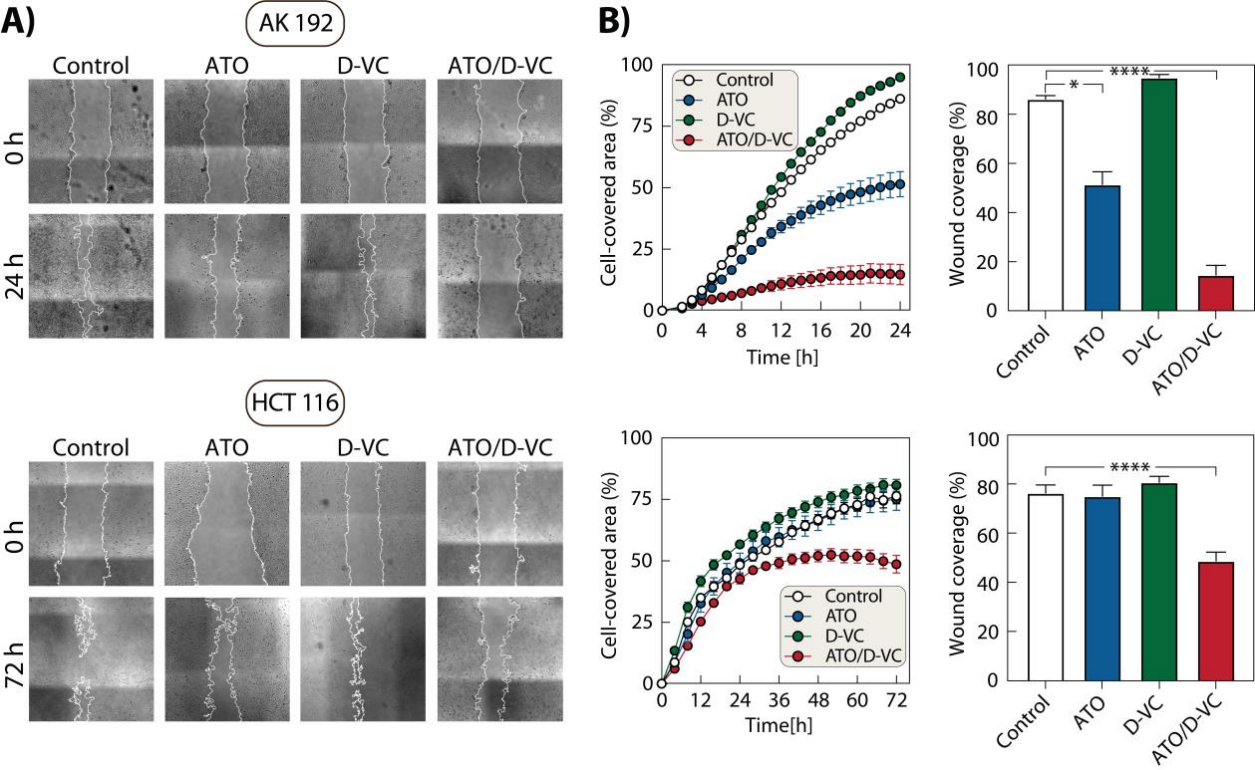
FIGURE 2: Scratch assay results of AK 192 and HCT 116 cells untreated and treated with either ATO, D-VC or their combination. **(A)** Representative images of each cell line and treatment at 0 h and final time points (24 h for AK 192, 72 h for HCT 116). Left panels show time-course of wound closure as % cell-covered area. **(B)** Right panels show final wound coverage at 24h (AK 192) and 72h (HCT 116). Results of three independent experiments are presented as mean ± SEM.) Statistical analysis was performed using the Kruskal-Wallis test for AK 192 cells and ordinary one-way ANOVA for HCT 116 cells. Stars indicate statistical significance: p<0.05 (*), p<0.01 (**), p<0.001 (***) and p<0.0001 (****).

Since ATO/D-VC treatment induces early apoptotic features without immediate cell death, we decided to assess whether remaining attached cells retain functional properties- particularly their ability to migrate (**Fig 2A**). The scratch assay revealed that migration of ATO/D-VC treated AK 192 cells was 6 times slower than that of control cells, and 1.6 times slower in HCT 116 cells. Interestingly, in both cell lines, the fastest wound coverage was observed under treatment with D-VC, which was 95% in AK 192 cells, followed by control cells covering 85.2% of the initial wound area. Similarly, D-VC treated HCT 116 cells closed 80.7% of the gap, compared to 76.4% in control cells. The ATO alone did not alter the migration of HCT 116 cells (75% closure), but impaired the migration of AK 192 cells (51.5% closure), making it 1.7 times slower than control (**Fig 2B**).

Moreover, we observed that ATO/D-VC treatment affected AK 192 cells’ migration immediately, resulting in slower migration from 4 h after treatment. Meanwhile, ATO/D-VC treated HCT 116 cells had a steady increase in migration within the first 24 h before reaching a plateau. This suggests cell line-specific kinetics in response to treatment, potentially reflecting differences in stress signalling or cytoskeletal dynamics.

### Cortical (cytoskeletal) stiffness of pre-apoptotic cells increases under ATO/D-VC treatment

**Figure 3:**
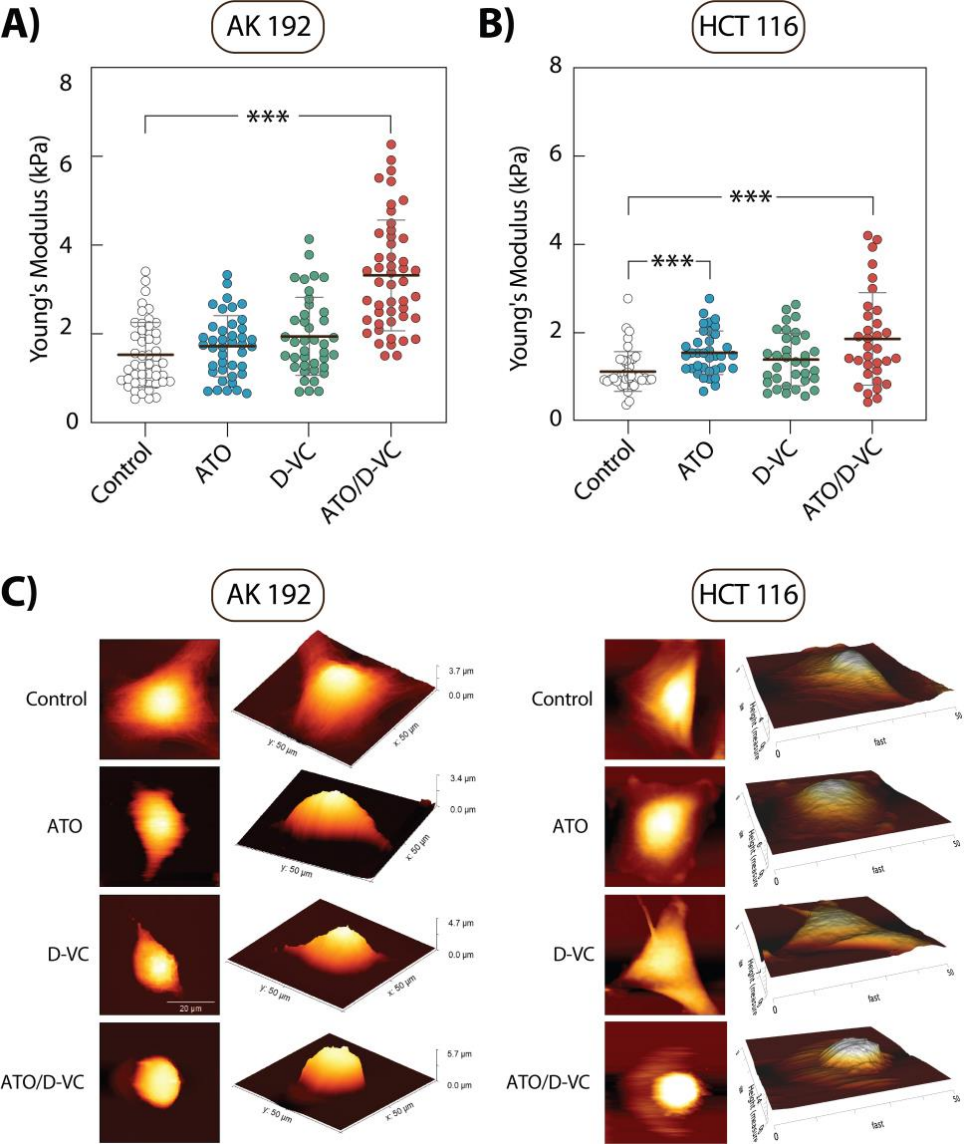
FIGURE 3: AFM measurements of cortical stiffness in AK 192 and HCT 116 cell lines. **(A)** Young's Modulus (cortical stiffness) of AK 192 cells for the control, ATO, D-VC, or their combination. **(B)** Young's Modulus of HCT 116 cells at 72 h under the same treatment conditions. Results of three independent experiments with mean stiffness calculated from measurements of at least 40 cells per condition. Statistical analysis performed using the Kruskal-Wallis test. Stars indicate statistical significance: p<0.05 (*), p<0.01 (**) and p<0.001 (***). C) Representative AFM high-resolution images of cellular cortex with corresponding 3D topographical structures showing cell surface morphology under different treatment conditions. Scale bar: 20 µm. kPa = kiloPascals.

Given that cytoskeletal dynamics and cell stiffness play critical roles in cell motility, we next measured the cortical stiffness of attached pre-apoptotic cells using atomic force microscopy (AFM). AK 192 cells treated with ATO/D-VC were 2.17 times stiffer (3.32 kPa) compared to controls (1.53 kPa). Cells treated with individual drugs, ATO (1.72 kPa) and D-VC (1.94 kPa), did not show significant differences from the control but were markedly softer than cells treated with the combined drugs (**Fig. 3A**). Similar trend was observed in HCT 116 cells, where the control stiffness was 1.13 kPa, and ATO/D-VC treatment increased it by 1.7-fold to 1.87 kPa. Treatment with ATO and D-VC alone increased stiffness to a lesser extent (1.55 kPa and 1.40 kPa, respectively) (**Fig. 3B**).

High-resolution AFM images revealed that control and single drug treated AK 192 and HCT 116 cells maintained an organized cytoskeletal structure, whereas the cytoskeleton appeared disrupted in cells treated with the combination of oxidizing drugs, resulting in a more rounded morphology (**Fig. 3C**). Together, these findings indicate that ATO/D-VC treatment increases stiffness of pre-apoptotic cells and alters their cytoskeletal morphology.

### Oxidative stress-induced cortical stiffening is actin-dependent, and reversible by partial depolymerization

To explore whether the observed changes in cortical stiffness are associated with alterations in F-actin content, we labelled filamentous actin with phalloidin staining and quantified total F-actin levels by flow cytometry. F-actin measurements were performed under standardized cell density conditions and normalized to control for potential cell number artifacts.

**Figure 4:**
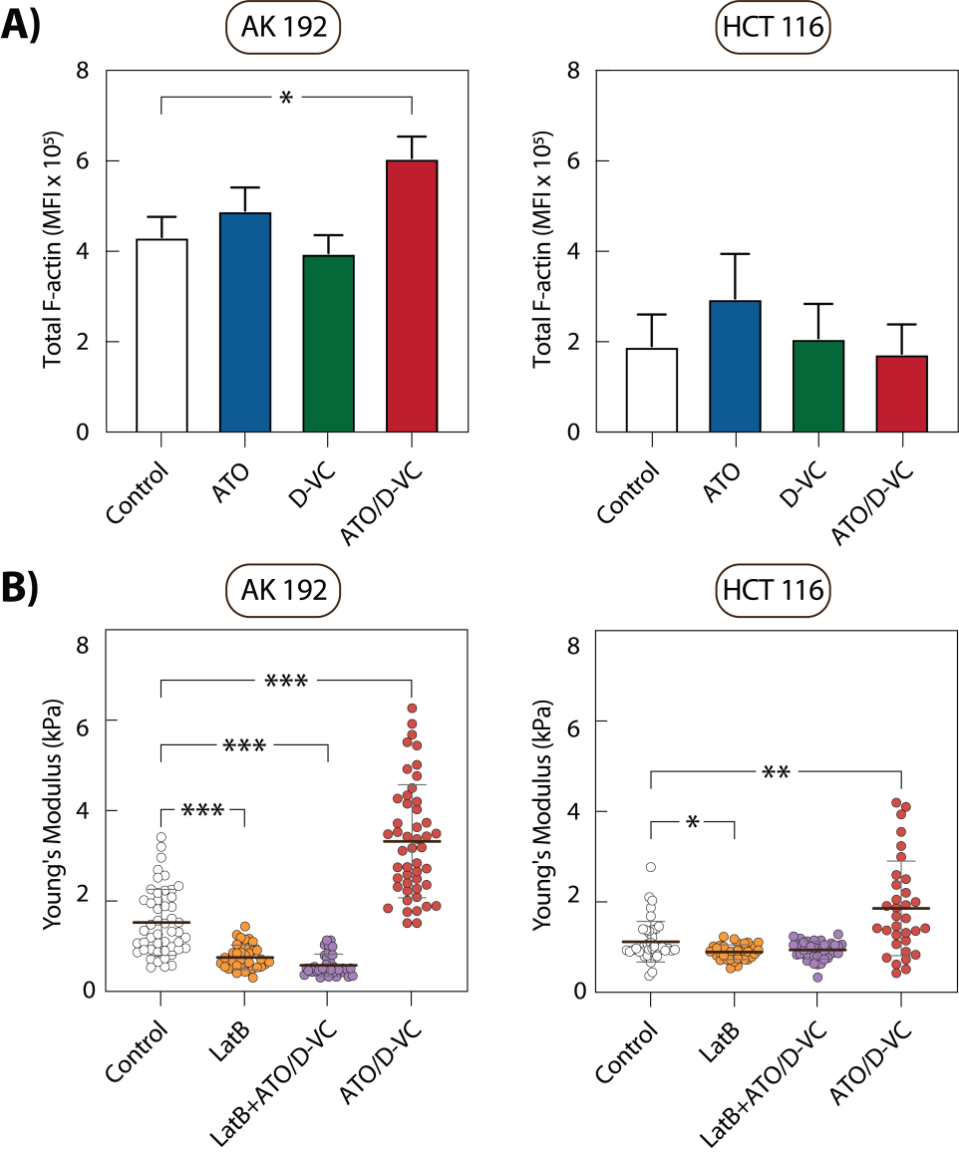
FIGURE 4: F-actin content and cortical stiffness modulation by oxidative stress treatments. **(A) **Mean Fluorescence Intensity (MFI) of total F-actin in AK 192 and HCT 116 cells treated with Control, ATO, D-VC, or ATO/D-VC combination for 24 h and 72 h respectively, measured by phalloidin staining and flow cytometry. **(B)** AFM measurements of cortical stiffness in AK 192 and HCT 116 cells following treatment with ATO/D-VC and/or Latrunculin B (LatB). Cells were treated with ATO/D-VC for 24 h (AK 192) or 72 h (HCT 116), followed by 30-minute treatment with 50 nM LatB prior to AFM measurements. Results represent three independent experiments with mean stiffness calculated from measurements of at least 40 cells per condition. F-actin measurements were acquired under matched cell density conditions with normalization applied to control for cell number variations. Statistical analysis performed using Kruskal-Wallis test, mean ± SEM. Stars indicate statistical significance: p<0.05 (*), p<0.01 (**) and p<0.001 (***).

In AK 192 cells, where ATO/D-VC treatment markedly increased stiffness (**Fig. 3A**), we observed a corresponding 1.4-fold increase in F-actin MFI. In contrast, HCT 116 cells showed increased stiffness under ATO/D-VC treatment without a detectable change in total F-actin levels. In both cell lines, single treatments with either ATO or D-VC did not change F-actin contents significantly, with a slight increase in F actin under ATO treatment (**Fig. 4A**).

Notably, under basal conditions, AK 192 cells displayed 2.29-fold higher total F-actin levels compared to HCT 116 cells, as well as 1.35-fold higher stiffness. This suggests a cell line-dependent correlation between total F-actin abundance in cells and their stiffness. However, under oxidative stress, global F-actin levels do not consistently reflect cortical stiffness changes—particularly in HCT 116 cells. These results suggest that changes in total F-actin content might not be the direct cause of cortical stiffness, rather, it might be regulated by an increase in F- actin localized in the cell apical cortex (the region that directly probed by the AFM tip).

To directly test whether the increased stiffness observed under ATO/D-VC treatment is dependent on the actin cytoskeleton, we next treated cells with Latrunculin B (LatB), a compound known to depolymerize F-actin. LatB at 50 nM was added post ATO/D-VC treatment 30 min before AFM measurements.

To identify a LatB concentration that partially reduces F-actin while preserving cytoskeletal structure, we measured F-actin intensity with flow cytometry. Based on that we selected 50 nM LatB, which reduced F-actin content by 29.3% in HCT 116 cells and 58.0% in AK 192 cells. Thus, LatB treatment alone reduced the stiffness of control AK 192 cells by 50.8% from 1.53 kPa to 0.75 kPa. ATO/D-VC-treated AK 192 cells exposed to LatB showed a further decrease to 0.58 kPa, making them significantly softer than either control or ATO/D-VC treated cells without LatB. In HCT 116 cells, LatB alone caused 20.3% reduction in stiffness, from 1.13 kPa to 0.90 kPa. In ATO/D-VC-treated HCT 116 cells, LatB further lowered stiffness to 0.94 kPa (**Fig. 4B**). Importantly, these values are lower than those observed under ATO/D-VC treatment alone, confirming that increase in stiffness in pre-apoptotic cells is dependent on F-actin localized in the apical cell cortex and can be reversed by moderate F-actin depolymerization. However, it should be noted that LatB disrupts both passive F-actin networks and active tension generation. This experiment only demonstrates actin-dependence but cannot distinguish whether ROS affects cortical stiffness through either increased F-actin polymerization, enhanced actomyosin contractility or increased the cell osmotic pressure.

### ROS mediates cortical stiffening and cytoskeletal remodelling, reversible by NAC

**Figure 5:**
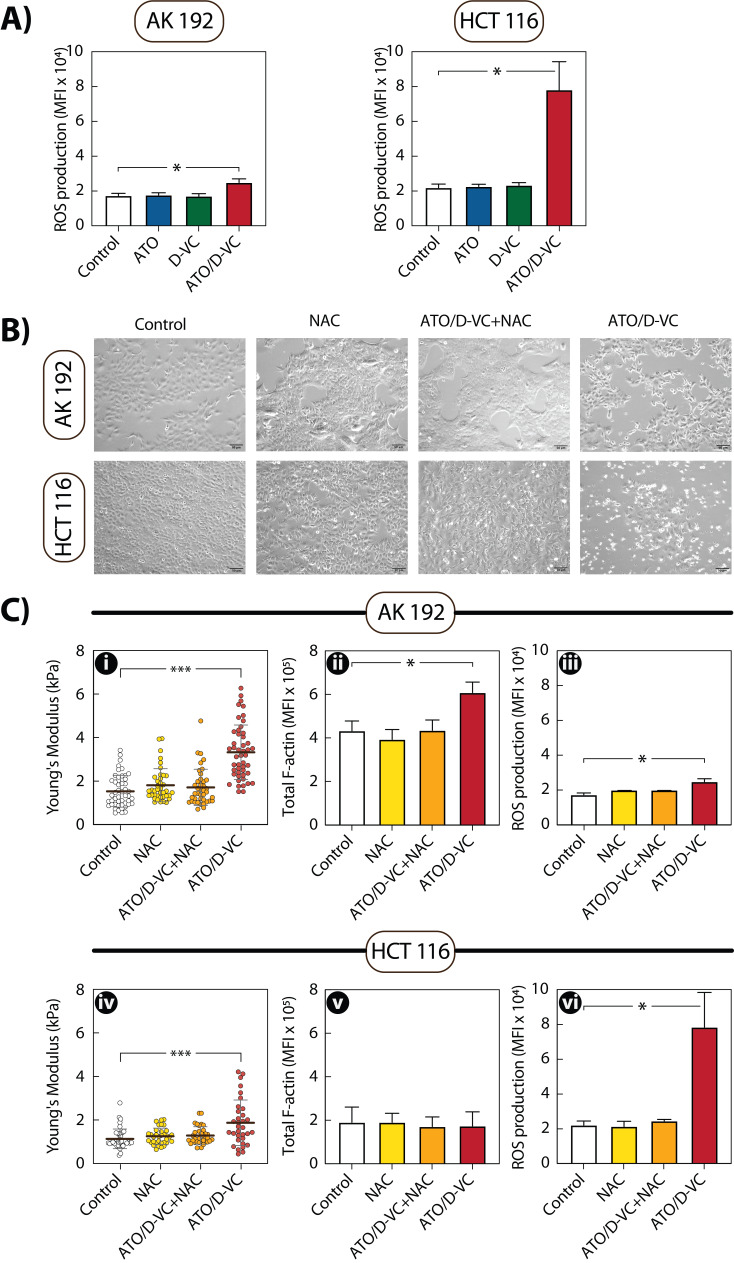
FIGURE 5: ROS production and its role in oxidative stress-induced cellular changes. **(A) **Mean Fluorescence Intensity (MFI) of mitochondrial ROS in AK 192 and HCT 116 cells treated with Control, ATO, D-VC, or ATO/D-VC combination, measured by MitoSox Red staining and flow cytometry. **(B)** Representative brightfield images showing morphological changes in NAC-treated cells. **(C)** Effect of ROS quenching by 1-hour pre-treatment with 5 mM N-acetylcysteine (NAC) followed by 24 h (AK 192) or 72 h (HCT 116) treatment with ATO/D-VC. Top row (AK 192): (i) AFM cortical stiffness measurements, (ii) total F-actin content by flow cytometry, (iii) mitochondrial ROS levels by MitoSox staining. Bottom row (HCT 116): (iv) AFM cortical stiffness measurements, (v) total F-actin content by flow cytometry, (vi) mitochondrial ROS levels by MitoSox staining. Results represent three independent experiments with mean ± SEM. For AFM measurements, at least 40 cells measured per condition. Statistical analysis performed using Kruskal-Wallis test. Stars indicate statistical significance: p<0.05 (*), p<0.01 (**) and p<0.001 (***).

Flow cytometry analysis with MitoSox staining was performed to estimate the production of mitochondrial ROS (mtROS) in AK 192 and HCT 116 cells. In both AK 192 and HCT 116 cells, single-drug treatments (ATO or D-VC) did not significantly increase mtROS levels. In contrast, combined ATO/D-VC treatment induced a notable rise in mtROS. In AK 192 cells, MFI increased by 1.43-fold from 17 486.7 in control cells to 24 950.8 under treatment. A more pronounced effect was observed in HCT 116 cells, where MFI increased 3.55-fold, from 22 048.2 in control cells to 78 189.5 following ATO/D-VC treatment (**Fig.  5A**).

To test whether ROS mediates observed cytoskeletal remodelling in pre-apoptotic cells, we quenched ROS with the antioxidant *N*-acetyl cysteine (NAC). Treatment with 5 mM NAC alone did not alter cell morphology. Moreover, 1 h NAC pre-treatment markedly protected cells from ATO/D-VC drug combination, reducing cell detachment and shrinkage compared to ATO/D-VC alone (**Fig. 5B**). This suggests a ROS-dependent mechanism underlying these cytotoxic morphological alterations.

Flow cytometry MitoSox analysis confirmed that NAC alone did not affect baseline mtROS levels in AK 192 cells. However, NAC pre-treatment before ATO/D-VC exposure significantly reduced mtROS production by 1.24-fold compared to ATO/D-VC alone (**Fig. 5C iii**). Significantly, this ROS quenching reversed the cytoskeletal changes previously induced by oxidative stress. F-actin content in NAC and ATO/D-VC-treated cells dropped by 1.4-fold compared to ATO/D-VC alone (**Fig. 5C ii**) and cortical stiffness decreased nearly twofold, from 3.319 kPa under ATO/D-VC to 1.704 kPa with NAC pre-treatment. Notably, NAC and ATO/D-VC values resemble the stiffness of control cells (1.526 kPa) (**Fig. 5C i**).

A more pronounced rescue effect was observed in HCT 116 cells, where NAC pre-treatment before ATO/D-VC exposure reduced mtROS production by 3.55-fold (**Fig. 5C vi**). This was accompanied by a 1.46-fold decrease in cortical stiffness, from 1.866 kPa under ATO/D-VC treatment to 1.277 kPa with NAC pre-treatment (**Fig. 5C iv**). As previously noted, (**Fig. 4A**), total F-actin levels in HCT 116 cells remained unchanged across conditions. The same was observed here - NAC and NAC with ATO/D-VC have similar F-actin content as in the control. (**Fig. 5C v**).

Together, these data suggest that ROS mediates observed changes in stiffness and cytoskeletal remodelling, and that scavenging ROS with NAC can attenuate this effect.

### Cytoskeletal remodelling and cortical stiffening are consistently induced by diverse ROS-generating agents

**Figure 6:**
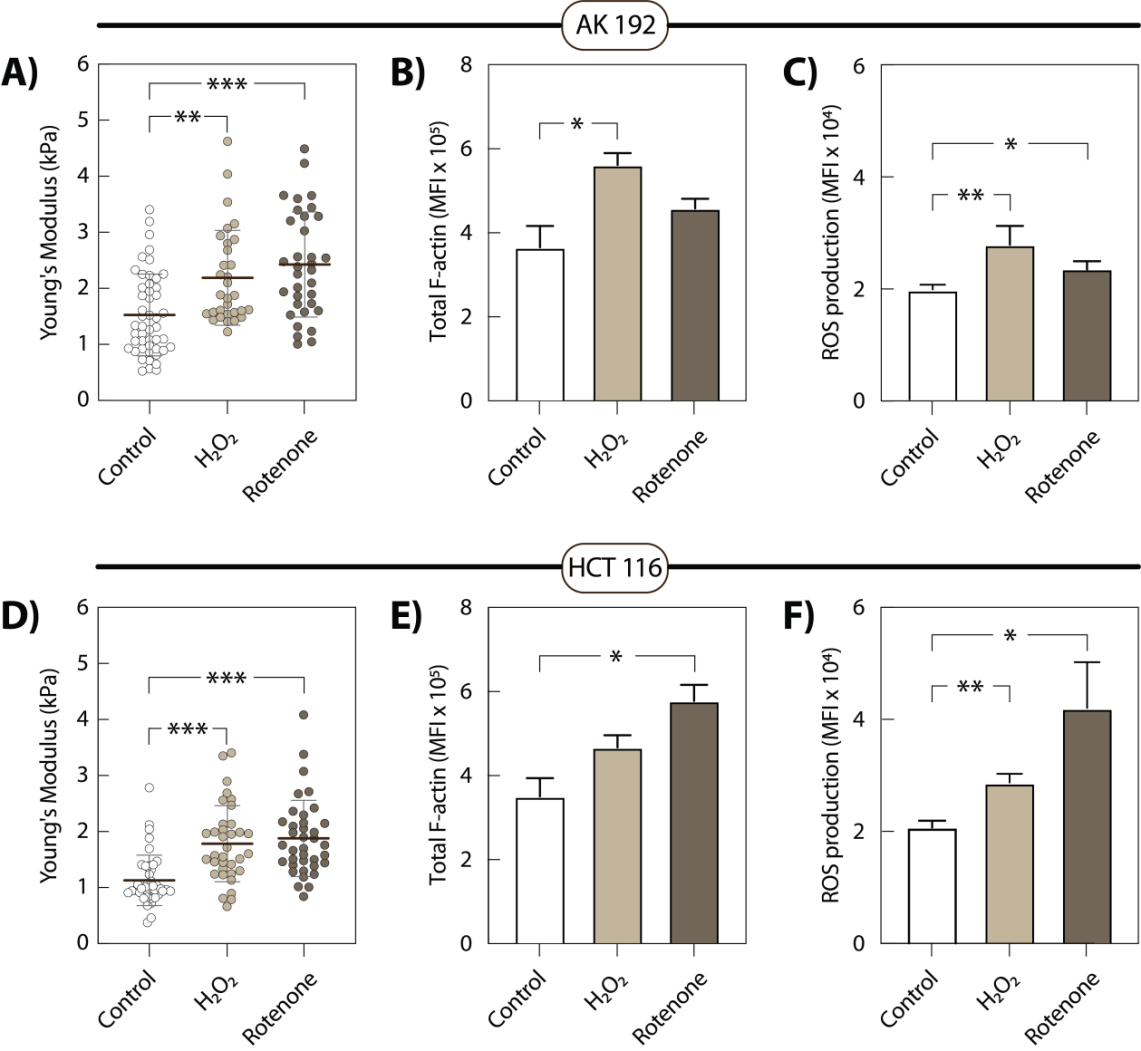
FIGURE 6: Effects of ROS-inducing treatments on AK 192 and HCT 116 cells. Cells were treated with 150 µM H_₂_O_₂_ for 1 h or 2.5 µM rotenone for 24 h. **(A, D)** AFM cortical stiffness measurements in AK 192 **(A)** and HCT 116 **(D)** cells under control conditions and following H_₂_O_₂_ or rotenone treatment. **(B, E)** Flow cytometry quantification of total F-actin content (Mean Fluorescence Intensity) in AK 192 **(B)** and HCT 116 **(E)** cells. **(C, F)** Mitochondrial ROS (mtROS) levels assessed by MitoSox staining and flow cytometry in AK 192 **(C) **and HCT 116 **(F)** cells. Results represent three independent experiments with mean ± SEM. For AFM measurements, at least 40 cells were measured per condition. Stars indicate statistical significance: p<0.05 (*), p<0.01 (**) and p<0.001 (***).

To investigate whether other ROS-inducing agents similarly affect the cytoskeleton and cortical stiffness, we used classical ROS inducers such as hydrogen peroxide (H_₂_O_₂_) and rotenone, an inhibitor of mitochondrial complex I. One hour treatment with 150 µM H_₂_O_₂_ induced a 1.42-fold increase in ROS production in AK 192 cells (MFI increased from 19 664 in control to 27 942 in H_₂_O_₂_-treated cells) (**Fig. 6C**). Similarly, H_₂_O_₂_ increased ROS by 1.38-fold in HCT 116 cells (MFI from 21 763.9 in control to 30 079.4 in treated cells) (**Fig. 6F**). This oxidative stress induced by H_₂_O_₂_ caused a 1.54-fold increase in total F-actin content in AK 192 cells, accompanied by a 1.44-fold increase in cortical stiffness (**Fig. 6A, B**). In HCT 116 cells, H_₂_O_₂_ treatment caused a 1.33-fold increase in F-actin content and a 1.58-fold increase in cortical stiffness (**Fig. 6D, E**).

Treatment with 2.5 *μ*M rotenone for 24 h exhibited 1.21-fold increase in ROS levels in AK 192 cells and a twofold increase in ROS in HCT 116 cells (**Fig. 6C, F**). Rotenone treatment also caused a 1.25-fold increase in total F-actin amount along with 1.60 times increase in stiffness in AK 192 cells (**Fig. 6A, B**). We also observed a 1.65-fold increase in F-actin as well as a 1.67-fold increase in stiffness in HCT 116 cells (**Fig. 6E, D**).

These results suggest that ROS levels elevation, regardless of the source, universally promotes increased F-actin content and cortical stiffening in pre-apoptotic cells, indicating a shared downstream effect of oxidative stress on the cell cytoskeleton.

## DISCUSSION

ROS at low levels are crucial for essential cellular functions such as proliferation, migration, and differentiation by activating signalling pathways like MAPK, WNT, and TGF-ß 16. However, excessive ROS levels become cytotoxic, damaging nucleic acids and proteins, leading to apoptosis. Cancer cells typically have higher basal levels of ROS compared to normal cells 17, where ROS act as signal-transducing messenger in many redox-sensitive molecular pathways involved in tumorigenesis 18,19,20. To survive this oxidative burden, cancer cells develop compensatory antioxidant mechanisms to maintain homeostasis. Some therapeutic strategies exploit this by inducing mtROS beyond the buffering capacity, leading to cell death.

In this study, we examined how oxidative stress induced by ATO and D-VC affects the biophysical properties of pre-apoptotic cancer cells. Combination of these drugs is known to impair mitochondria and induce suicidal ROS production in cancer cells 14,15. Our focus was on the fate of pre-apoptotic AK 192 and HCT 116 cells and changes in their cortical stiffness, cytoskeletal organization, and migration following treatment.

The combined ATO/D-VC treatment, which has been shown to act synergistically, increased the number of pre-apoptotic cells, reduced proliferation, and impaired migration in AK 192 cells after 24 h and in HCT 116 after 72 h. While re-duced migration was partly linked to lower proliferation, it was also correlated with increased cortical stiffness. Prior studies showed that stiffer cancer cells migrate more slowly due to impaired deformation, reduced detachment from surfaces and higher force generation required for movement 5,21,22. Consistent with this, our AFM measurements revealed a 2-fold increase in cortical stiffness and a 6-fold reduction in migration in AK 192 cells; 1.7-fold higher stiffness and 1.6-fold slower migration in HCT 116 cells. Single drug application (ATO or D-VC) did not induce these changes, suggesting a synergistic effect of treatment.

Since the cell cortex consists mainly of actin filaments 23, it was hypothesized that F-actin polymerization might cause the observed significant increase in cortical stiffness. Quantification of total F-actin using flow cytometry revealed a cell line-dependent relationship between total F-actin levels and cortical stiffness under basal conditions, with AK 192 cells exhibiting both higher F-actin content and greater stiffness compared to HCT 116. However, under oxidative stress, this relationship was no longer consistent: in AK 192 cells, increased stiffness was accompanied by a rise in total F-actin, while in HCT 116, stiffness increased despite unchanged F-actin levels. This suggests that the total F-actin content alone does not fully explain cortical mechanics, which is consistent with recent findings 10. Importantly, cortical stiffness depends not only on F-actin content but also on the active contractile state of the actin-myosin network. ROS-induced changes in calcium signalling or myosin activity could increase cortical tension without necessarily increasing total F-actin levels, providing an alternative explanation for the stiffness increases observed in AK 192 and HCT 116 cells. Instead, we assumed that cell stiffness may be more dependent on the organization or localization of F-actin—particularly within the apical cortex directly probed by AFM—rather than its overall abundance.

To test the dependence on actin, we treated cells with 50 nM LatB for 30 min to partially depolymerize F-actin 24. Moderate depolymerization of actin by 30-50% with LatB reduced the stiffness of ATO/D-VC-treated cells to near-control levels in both cell lines, confirming that increased cortical stiffness is dependent on F-actin in the apical cortex.

While combined ATO/D-VC treatment elevates ROS levels, we hypothesized that ROS drives the observed increase in cellular stiffness and cytoskeletal remodelling. To test this hypothesis, we quenched ROS with NAC, which restored ROS levels to baseline and reversed the stiffening effect, thereby implicating ROS is a mechanistic trigger for these cellular changes. To determine whether this ROS-mediated stiffening effect is universal, we treated cells with alternative ROS-generating agents: 150 µM exogenous H_₂_O_₂_ and 2.5 µM rotenone. These concentrations fall within ranges commonly reported to induce moderate oxidative stress 24,25. Treatment with these ROS inducers significantly increased total F-actin content and cortical stiffness compared to controls, further confirming the role of oxidative stress as a driver of cytoskeletal remodelling and mechanical property changes.

Oxidative stress and ROS can affect the actin cortex via multiple mechanisms. These mechanisms could affect both passive actin network properties (through polymerization and crosslinking) and active contractile forces (through calcium signaling and myosin activation). It can also activate Rac1 and RhoA GTPases, leading to actin polymerization via the ARP2/3 complex and ROCK signaling, respectively 13,26,27. ROS may also induce the formation of free barbed ends on actin filaments, accelerating their polymerization 28,29. The resulting actin network densification in the apical cortex leads to stiffening of the cell cortex, which results in slower migration. Our finding is significant in the context of a long-standing debate about how ROS influence F-actin dynamics. While some studies report ROS-induced actin polymerization, others describe depolymerization via S-glutathionylation of the Cys374 residue 11,30 or inhibition of actin-profilin interaction 31.

Our findings provide new mechanistic insights on how ROS selectively remodels cortical actin to control cancer cell stiffness and migration. By demonstrating that cortical, rather than total, F-actin drives ROS-induced stiffness changes, we resolve the apparent controversy between studies showing ROS-induced actin polymerization versus depolymerization. Understanding this spatial selectivity has important implications for how oxidative stress affects cancer cell behavior and may inform therapeutic strategies targeting the mechanical properties of cancer cells through pro-oxidant treatments. This distinction between total and cortical F-actin effects resolves an apparent contradiction in the literature, where some studies report ROS-induced actin polymerization while others report depolymerization. Our findings suggest that the subcellular localization of actin changes, rather than global actin content, determines the mechanical consequences of oxidative stress.

Despite demonstrating that F-actin polymerization contributes significantly to oxidative stress-induced cortical stiffening, we acknowledge that actin is not the sole target. ROS affect a broader network of cytoskeletal and membrane-associated proteins, including actin regulators (cofilin, profilin, Arp2/3) and crosslinkers (filamin A, α-actinin, ERM proteins) 32. These components act together to shape the overall mechanical response. Further studies are needed to examine which key regulatory proteins and posttranslational modifications of actin are affected by ROS, in order to fully elucidate the interaction between oxidative stress and actin. Also, additional studies using myosin and Rho-kinase inhibitors (e.g., blebbistatin and Y27632) alongside actin depolymerizing agents would help distinguish whether ROS-induced stiffening primarily results from increased passive F-actin networks or enhanced active tension generation.

Finally, we demonstrate that elevated ROS levels trigger actin polymerization in the apical cell cortex, resulting in increased cortical stiffness and a corresponding reduction in cell migration. This finding is significant, as the connection between oxidative stress and cellular stiffness had previously remained unclear.

## MATERIAL AND METHODS

### Cell culture

Two cancer cell lines were used in the study: the mouse pancreatic AK 192 and the human colon cancer HCT 116 cell lines. The AK 192 cell line was provided from the original sources by Dr. Haoqiang Ying and HCT 116 cell line was purchased from ATCC (https://www.atcc.org/products/ccl-247). All cells were passaged and grown until they reached a confluency of 80%. Cells were grown in Dulbecco's Modified Eagle Medium (DMEM, US Biological #D9807) supplemented with 5% fetal bovine serum (FBS), *L*-glutamine, and 1% Penicillin/Streptomycin (Pen/Strep) in the incubator at 37°C with 5% CO_2_ supply. Throughout all experiments, Doxycycline was added to AK 192 cells at a concentration of 2mg/ml, to induce KRAS mutation in cells by activating the promoter.

### Drug treatments

Cells were seeded at appropriate densities for various experiments and left to adhere for 24 h. AK 192 cells were treated for 24 h with ATO (Sigma #A1010) at 5 µM and D-VC (Sigma #856061) at 1 mM, either as a single drug (ATO or D-VC) or their combination. HCT 116 cells were treated with ATO at 6 µM and D-VC at 1.25 mM for 72 h. Control cells were maintained in DMEM for the same length of time. LatB (Sigma #428020) was added at 50 nM 30 min prior to AFM measurements. The drug was not washed out during the experiment to avoid reversible actin polymerization. NAC (Sigma A7250-50G) was added 1 h before treatment with oxidative drugs, at a final concentration 5 mM. The cells were treated with H_₂_O_₂_ (Honeywell #31642-500HL) for 1h at 150 µM. A fresh 8.82 mM intermediate stock was prepared from an 882 mM H_₂_O_₂_ stock using ultrapure water before each experiment. Rotenone (Sigma #R8875) was used at a concentration 2.5 µM for 24 h.

### Microscopic Observation of Cell Morphology

Both cell lines were cultured in 6-well plates for 24 h to adhere. The cells were further incubated with oxidative drugs for 1 h, 24 h or 72 h, and cell morphological changes were observed and imaged by a phase contrast microscope Primovert (Zeiss) using a 10X objective.

### Annexin V/PI apoptosis assay

AK 192 and HCT 116 cells were cultured in 6-well plates until 80% confluency and treated with the indicated concentration of single drugs ATO, D-VC, or their combination for 24 h and 72 h respectively. Following treatment, the cells were harvested by gentle trypsinization, washed with PBS, and resuspended at 1.000.000 cells per mL in annexin binding buffer. 150 µL of cells suspension was stained with 3 µL Annexin V FITC and 1 µL PI (both from ThermoFisher, A23204, and P3566 respectively) and incubated in the dark for 15 min at RT. The stained cells were resuspended with an additional 400 µL of annexin binding buffer and minimum 30 000 events acquired with flow cytometry.

### Cell proliferation assessment with CFSE staining

AK 192 and HCT 116 cells were centrifuged and gently resuspended in pre-warmed PBS containing 5 µM carboxyfluorescein succinimidyl ester (CFSE; ThermoFisher, #C34554). Cells were incubated for 20 min at 37°C, then centrifuged and resuspended in pre-warmed DMEM. Approximately 30 000 cells were acquired by flow cytometry to assess initial dye intensity. The remaining CFSE-stained cells were counted and seeded in 6-well plates at densities of 200 000 (AK 192) and 250 000 (HCT 116) cells per well. After 24 h of adherence, cells were treated with oxidative agents. Following treatment, cells were trypsinized and analyzed by flow cytometry. A decrease in CFSE intensity indicates increased cell proliferation due to dye dilution during cell division.

### Mitochondrial ROS quantification using MitoSOX

Superoxide production was measured with MitoSox (from ThermoFisher, M36008). Cells were cultured in 6 well plates, collected and 500.000 cells were stained with 2.5 µM of MitoSox for 30 min at 37°C, washed, and resuspended in PBS. The amount of ROS was quantified presented as mean fluorescent intensity (MFI) of MitoSox. A minimum of 30.000 events were acquired on the Attune NxT Flow Cytometer (ThermoFisher Scientific).

### F-actin quantification with phalloidin

AK 192 (30,000 cells/well) and HCT 116 (50,000 cells/well) were seeded in 24-well plates. Following treatment with oxidative agents, cells were fixed in 200 µL of 4% paraformaldehyde (PFA) for 15 minutes at room temperature (RT), then washed three times with PBS. Cells were permeabilized with 0.1% Triton X-100 for 10 minutes at RT, followed by another three PBS washes.

Next, cells were stained with 150 nM Phalloidin Alexa Fluor 488 (ThermoFisher A12379) in 1% BSA for 30 minutes at RT, washed with PBS, and subsequently trypsinized. The trypsinization was neutralized using 400 µL of PBS containing 1% BSA. A minimum of 30,000 events were acquired by flow cytometry, and F-actin content was quantified from phalloidin channel and reported as mean fluorescence intensity (MFI). Data were processed and normalized using FlowJo software. To control for potential cell density artifacts, experiments were performed using standardized seeding densities with explicit normalization to account for cell number variations between conditions.

### Atomic force microscopy (AFM)

For AFM experiments with oxidative drugs, AK 192 and HCT 116 cells were seeded in 35 mm culture dishes at densities of 200,000 and 250,000 cells per dish, respectively, in 2  mL of DMEM. Control conditions were plated at a lower density of 10,000 cells per dish to avoid overcrowding since control cells continued proliferating and did not undergo cell death like the treated groups. Post-treatment and before measurement on AFM, Hoechst 33342 (H21486) was added at a concentration of 1 µg/mL and incubated for 20 minutes in darkness. After that, the old media was replaced. For AFM imaging only single cells were measured.

To measure the cortical stiffness of cells, AFM JPK NanoWizard 4XP was used. After calibration, the tip had a spring constant of 0.2-0.4N/m and the final parameters for measurement of cortical stiffness of cells on AFM were: setpoint- 0.5nN; Z length- varies; Z speed- 30 µm/s; Contact time- 0s. AFM images for measurements of stiffness and height had a constant resolution of 1000 µM/pixel. Resolution for high-resolution images was constant- 312.5 µM/pixel.

AFM results were analyzed using JPKSPM Data Processing software. While we scanned the entire cell surface, Young’s modulus (stiffness) values were calculated from force-indentation curves measured at the highest point of each cell, using the Hertz model for a pyramidal tip.

### Scratch assay 

AK 192 cells were seeded at a density of 70.000 and HCT 116 cells at 300.000 per well in a 24-well plate. Cells were left to adhere for 24 h and when cells reached 90% monolayer confluency, the scratch was made using a sterile 200 µL pipette tip after careful wash a fresh DMEM with drugs was added to the respective wells, and plates were kept in an incubator with CytoSMART Omni system (Axion Biosystems) for time-lapse scanning of the wound area. Snap images of each well were taken every hour and further analysed using ImageJ plugin 33 . The percentage of scratch gap area was estimated by adjusting the variance windows radius, threshold value, and percentage of saturated pixels in the plugin.

### Statistical analysis 

Collected data were analyzed using the GraphPad prism 9 program. Depending on the results distribution one-way ANOVA test was used for normally distributed variables and a non-parametric test such as Kruskal-Wallis or Mann-Whitney for variables with sewed distribution. Levels of significance were set at a *p*-value less than 0.05.

## CONFLICT OF INTEREST

The authors declare no competing interests.

## AUTHOR CONTRIBUTION 

AJ- Initial conceptualization, experimental work, figures prepara-tion, writing original draft; AB- Investigation, experimental work, writing draft; AT experimental work, figure preparation, review of the draft; NR- experimental work, review of the draft; FM - review and editing draft, grant acquisition; TP- study design, review and editing draft, supervision; ANB- study design, writing original draft, review and editing, supervision, coordination of submission, corresponding author; DDS- study design, review and editing draft, supervision and grant acquisition. All authors contributed to the manuscript writing.
